# Supplementary material for: Nanobodies against SARS-CoV-2 reduced virus load in the brain of challenged mice and neutralized Wuhan, Delta and Omicron Variants
Source: bioRxiv. 2023 Mar 14:2023.03.14.532528. Preprint. [Version 1] doi: 10.1101/2023.03.14.532528 (PMC10054972; doi:10.1101/2023.03.14.532528)
Supplement: 1 [file NIHPP2023.03.14.532528V1-supplement-1.pdf]

bioRxiv preprint doi: https://doi.org/10.1101/2023.03.14.532528; this version posted March 14, 2023. The copyright holder for this preprint (which was not certified by peer review) is the author/funder, who has granted bioRxiv a license to display the preprint in perpetuity. It is made available under aCC-BY 4.0 International license.

Supporting Information

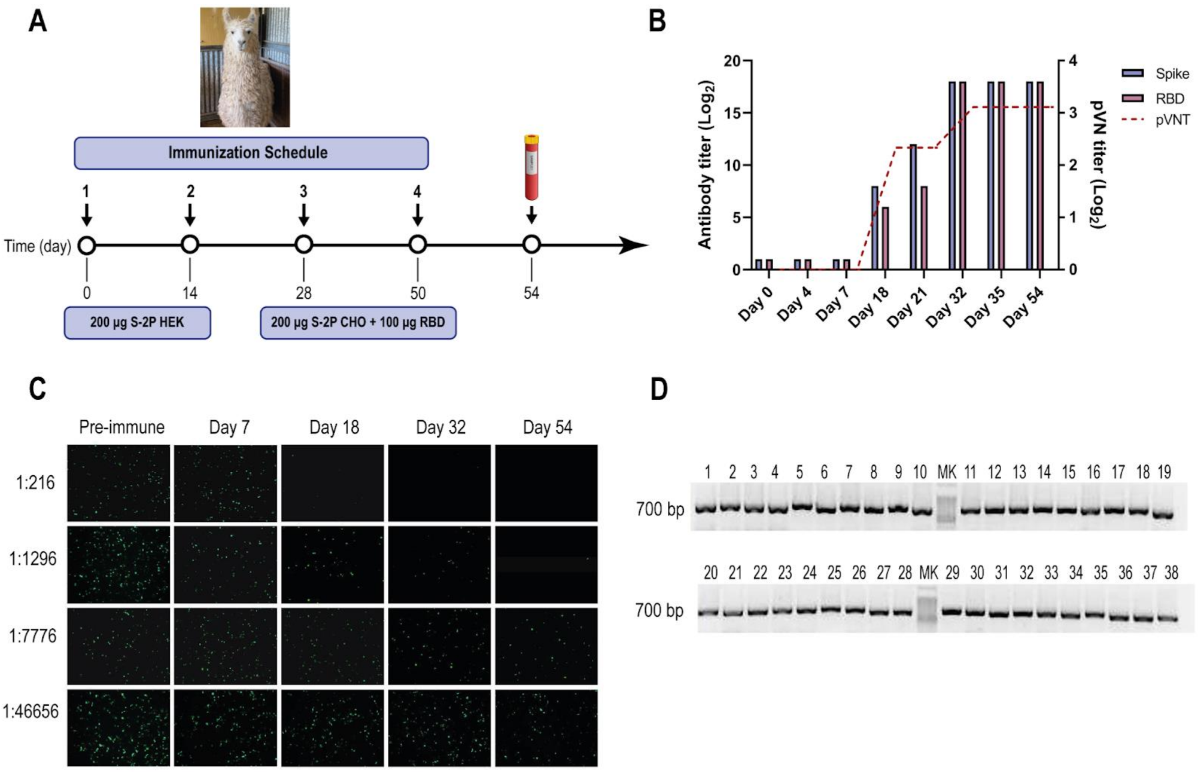

S1 Fig. SARS-CoV-2 llama immunization, immune response and Nb-library construction. (**A**) Immunization schedule: a llama was injected intramuscularly on days 0 and 14 with 200 µg of SARS-CoV-2 S-2P protein produced in HEK-293T and on days 28 and 56 with 200 µg of SARS-CoV-2 S-2P produced in CHO cells and 100 µg of RBD protein emulsified in Freund's adjuvant. Four days after the last boost, 200 ml of blood was collected, and peripheral lymphocytes were isolated to produce an immune library. (**B**) Total IgG titer determined by ELISA and neutralizing Ab titer determined by pVNT induced 4 and 7 days after each immunization. Four days after the third immunization (PID 32) a maximal antibody response was reached. (**C**) Picture illustrating neutralizing activity in llama serum determined by pVNT. The neutralization capacity increased after each immunization and correlated with a decrease in the number of fluorescent cells. A higher neutralizing titer was detected for a dilution of 1:1296 at PID 54. (**D**) Analysis of PCR products by agarose gel electrophoresis to confirm the number of transformants that had an insert of the proper size, each of the 48 clones that were randomly selected contained a genuine Nb fragment (~700 bp).

bioRxiv preprint doi: https://doi.org/10.1101/2023.03.14.532528; this version posted March 14, 2023. The copyright holder for this preprint (which was not certified by peer review) is the author/funder, who has granted bioRxiv a license to display the preprint in perpetuity. It is made available under aCC-BY 4.0 International license.

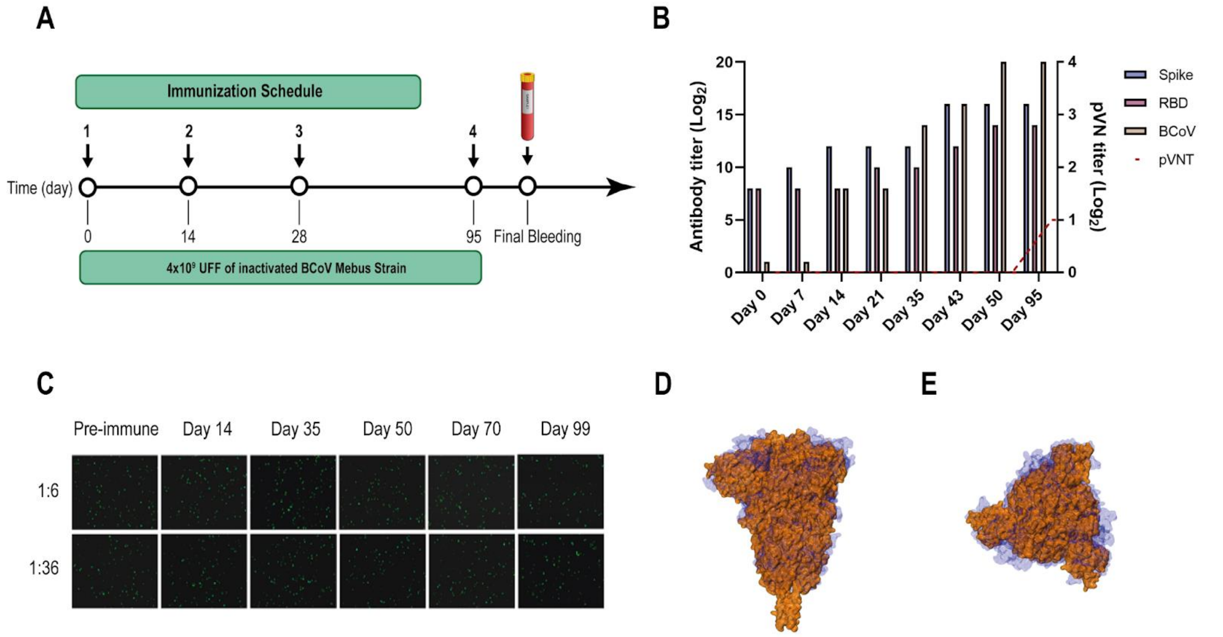

S2 Fig. BCoV Mebus llama immunization and immune response. (**A**) Immunization schedule followed to produce the Nb immune library: a llama was injected intramuscularly on days 0, 14, 28 and 95 with 4.00×109 UFF of the inactivated BCoV Mebus strain in Freund's adjuvant. Peripheral lymphocytes were isolated from 200 ml of blood collected four days after the final boost to generate the immune library. (**B**) Total IgG titer determined by ELISA for BCoV and SARS-CoV-2 RBD and S-2P proteins. (**C**) Picture showing non-neutralizing activity of sera from a llama immunized with BCoV Mebus against SARS-CoV-2 determined by pVNT. Superimposition of the S protein structures from SARS-CoV-2 (orange) and BCoV Mebus (blue) using VMD software. Frontal (**D**) and upper (**E**) view.

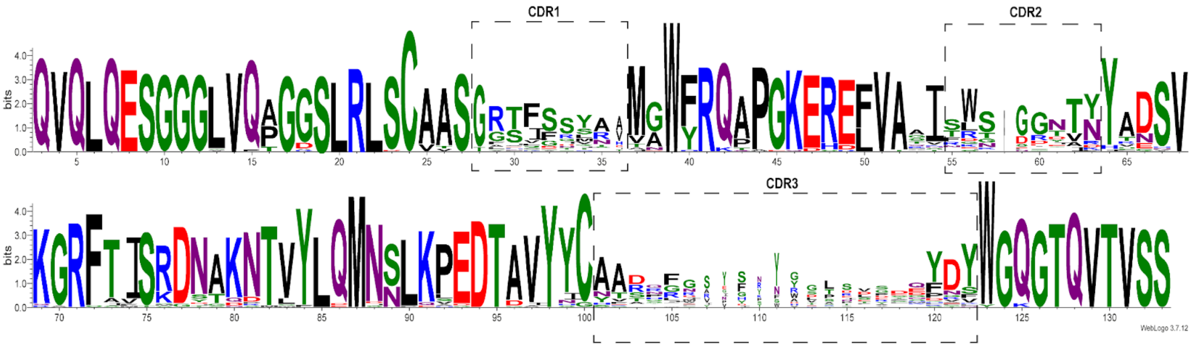

S3 Fig. Sequence Logo Plot of a diverse repertoire of SARS-CoV-2 Nbs. Logo representation of amino acid multiple sequence alignments of the 43 unique Nbs selected after biopanning with RBD or S-2P proteins. The height of symbols indicates the relative frequency of each amino acid at that position.

bioRxiv preprint doi: https://doi.org/10.1101/2023.03.14.532528; this version posted March 14, 2023. The copyright holder for this preprint (which was not certified by peer review) is the author/funder, who has granted bioRxiv a license to display the preprint in perpetuity. It is made available under aCC-BY 4.0 International license.

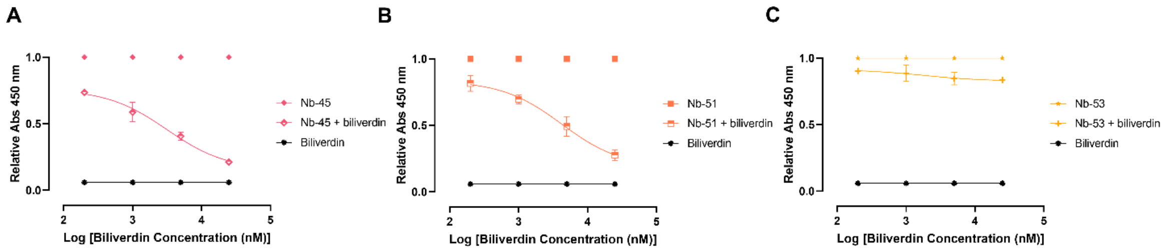

S4 Fig. Biliverdin decreases the binding of non-RBD binders to S-2P protein. Relative dose-response curves for Nb-45 (**A**), Nb-51 (**B**) and Nb-53 (**C**) in the absence or presence of different concentrations of biliverdin.

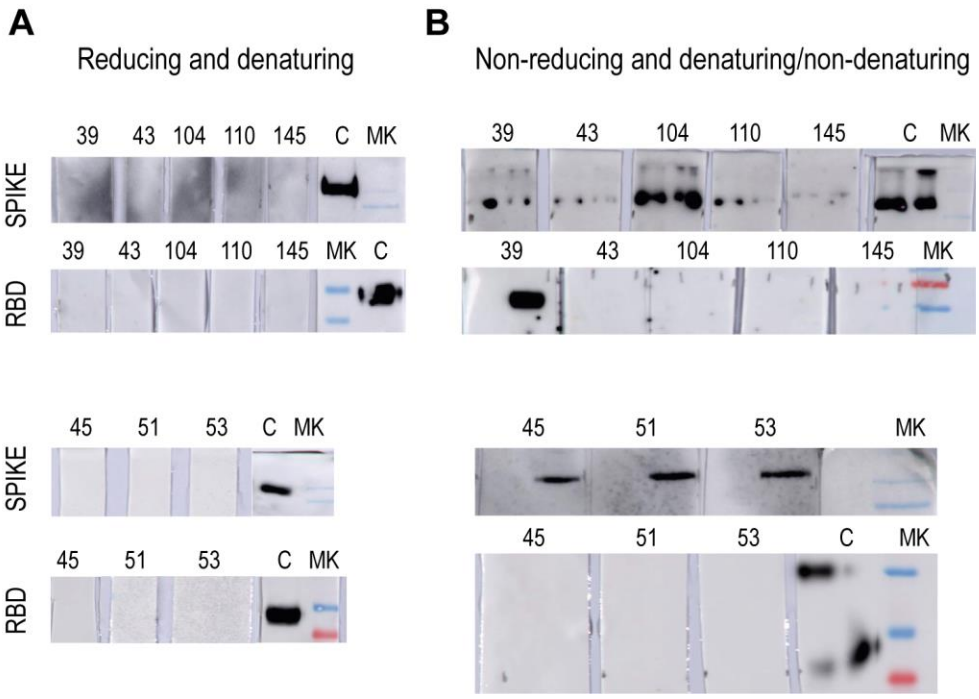

S5 Fig. Determination of Nanobodies binding to conformational or lineal epitopes by Western blot analysis. Different conditions of sample buffer and gel composition were tested to determine the Nbs reactivity. (**A**) Reactivity of Nbs to S-2P and RBD proteins under reducing and denaturing conditions. (**B**) Nanobodies recognition of S-2P and RBD proteins under non-reducing and denaturing (left well) or non-denaturing conditions (right well) for each Nbs. As can be observed, Nb-43, Nb-104, Nb-110 and Nb-145 can detect the S-2P protein only under non-denaturing conditions while Nb-39 recognizes not only S-2P but also RBD. Nanobody 45, Nb-51 and Nb-53 detect only the S-2P protein under non-denaturing conditions and do not bind to RBD.

bioRxiv preprint doi: https://doi.org/10.1101/2023.03.14.532528; this version posted March 14, 2023. The copyright holder for this preprint (which was not certified by peer review) is the author/funder, who has granted bioRxiv a license to display the preprint in perpetuity. It is made available under aCC-BY 4.0 International license.

1236 **S1 Table. Summary of HADDOCK results for the best Nanobody-Protein complexes**.

Ligand	HADDOCK score	VdW energy	Electrostatic energy	Desolvation energy	Buried Surface Area
**Biliverdin**	-40.4	-29.2	-86.9	-2.5	716.8
**Nb_43**	-48.1	-47.2	-176.4	3.5	1474.7
**Nb_45**	-63.7	-92.6	-259.2	-19.3	2374.6
**Nb_53**	-48.7	-93.3	-177.7	-19.9	2388.6

1237
